# Early social communication through music: State of the art and future perspectives

**DOI:** 10.1016/j.dcn.2023.101279

**Published:** 2023-07-27

**Authors:** Trinh Nguyen, Erica Flaten, Laurel J. Trainor, Giacomo Novembre

**Affiliations:** a Neuroscience of Perception and Action Lab, Italian Institute of Technology, Rome, Italy; b Department of Psychology, Neuroscience and Behavior, McMaster University, Hamilton, Canada; c McMaster Institute for Music and the Mind, McMaster University, Hamilton, Canada; d Rotman Research Institute, Baycrest Hospital, Toronto, Canada

**Keywords:** Social interaction, Musicality, Pitch, Rhythm, Development, Singing

## Abstract

A growing body of research shows that the universal capacity for music perception and production emerges early in development. Possibly building on this predisposition, caregivers around the world often communicate with infants using songs or speech entailing song-like characteristics. This suggests that music might be one of the earliest developing and most accessible forms of interpersonal communication, providing a platform for studying early communicative behavior. However, little research has examined music in truly communicative contexts. The current work aims to facilitate the development of experimental approaches that rely on dynamic and naturalistic social interactions. We first review two longstanding lines of research that examine musical interactions by focusing either on the caregiver or the infant. These include defining the acoustic and non-acoustic features that characterize infant-directed (ID) music, as well as behavioral and neurophysiological research examining infants’ processing of musical timing and pitch. Next, we review recent studies looking at early musical interactions holistically. This research focuses on how caregivers and infants interact using music to achieve co-regulation, mutual engagement, and increase affiliation and prosocial behavior. We conclude by discussing methodological, technological, and analytical advances that might empower a comprehensive study of musical communication in early childhood.

## Highlights

• Caregivers universally use music to communicate with their children.
• Children are biologically prepared to process musical pitch and rhythm.
• Social communication through music has been minimally investigated thus far.
• This research field can benefit from multi-person, home, and online paradigms.
• We discuss further methodological, technological, and analytical advances.

## 1 Introduction

It has long been noted that music-making is universal across human cultures (Huron, 2003, Mehr et al., 2019, Savage et al., 2021, Trainor, 2015). Likewise, engaging infants through song and rhythmic movement also appears to be universal (Trehub, 2019). This raises questions about the origins of the abilities that enable music-making, known collectively as *musicality*, the potential beneficial functions of music, and interactions between evolutionary pressures and cultural creation (Honing, 2018, Savage et al., 2021, Trainor, 2015).

One of the most comprehensive and widely accepted theories is that the most important functions of music relate to making music with others and to the social bonding that ensues from musical interactions (Savage et al., 2021). Group cohesion and affiliation between group members have obvious advantages in facilitating cooperation within a group, contributing to members’ self-identity, and distinguishing members who are within versus outside one’s group (Freeman, 1998). The origins and functions of musicality have also been considered through a developmental lens, particularly in relation to understanding why singing to infants is widespread, if not universal, across cultures (Cirelli et al., 2018, Mehr et al., 2019, Mehr et al., 2021). The social and emotional bonds between mothers (or primary caregivers) and infants are crucial for infants to survive and thrive physically, cognitively, emotionally, and socially (Feldman, 2017). Here we explore the role of music in fostering these bonds (Cirelli et al., 2018, Dissanayake, 2008). We examine the early musicality of infants, the musical behaviors of caregivers, and the nature, importance, and consequences of musical caregiver-infant interactions.

From before birth, infants are responsive to music (Edalati et al., 2023, Kisilevsky et al., 2004, Loewy et al., 2013). Young infants appear to be biologically prepared to process pitch and rhythmic structure while, at the same time, their musicality develops according to the specific musical system they are exposed to in their culture (Hannon and Trainor, 2007, Trainor and Hannon, 2013). Furthermore, infant-directed (ID) singing affects infants emotionally and can help them to regulate their state (Cirelli and Trehub, 2020). In tandem, parents seem to instinctively use music while interacting with their infants (Ilari, 2005), again suggesting that this is a biologically prepared behavior that may benefit parents and infants. Although most studies have examined either the development of infants’ musicality or the nature of the musical environment provided by parents, research indicates that infants and caregivers engage in mutually adaptive interactions during music-making, showing attunement and sensitivity to each other’s cues, which likely contributes to their emotional bonding and social affiliation (e.g., Markova et al., 2019; Savage et al., 2021).

The field is just beginning to understand the intricate complexities of these interactions, including coordination and communication between infants and caregivers engaged in music at physiological (Cirelli, Jurewicz et al., 2020), hormonal (Shenfield et al., 2003), neural (Markova et al., 2019), and behavioral (e.g., gaze/head orientation, movements, and expressions of affect) levels (Feldman, 2017). In musical interactions in early childhood, by *coordination* we refer to any temporal relationship between the behavior or physiological state of multiple individuals. This includes simple instances such as mere co-occurrence, e.g. gaze of the mother associating with arousal in the infant. Coordination can also include more complex instances, such as *synchrony*, which refers to rhythmic patterns exhibiting either period-coupling, as when two musicians play at the same tempo, or phase-coupling, as when actions are produced at the very same time or zero-phase. The umbrella term *coordination* does not necessarily require either tempo- or phase-coupling. *Communication*, on the other hand, refers to the exchange of information between multiple individuals. In the context of musical interactions, communication often involves conveying emotional and/or social information between a caregiver and an infant. Notably, coordination and communication can sometimes overlap in the context of musical interactions. For instance, when a parent sings a lullaby to a child to calm them (communication), they also rock the baby in time with the music (coordination).

We explore these ideas in the following sections and end by considering new research tools that can transform our knowledge in these domains. Section 2 will discuss the acoustic and non-acoustic features and implications of ID music, focusing on the caregiver’s perspective. Next, Section 3 describes early music processing in infants on the two main facets of music examined in the literature, pitch- and timing-related processing. In addition, we will elaborate on the effects of music on infants’ attention and regulation. Section 4 details the current state of the art of research on early musical interactions. The communicative functions examined are clustered into three subsections, namely co-regulation, prosocial behavior, and movement/mutual engagement. Section 5 describes future perspectives on how we can decode early musical interactions. We provide suggestions and challenges regarding suitable paradigms and the use of new and advanced analytical tools. Finally, we provide a brief conclusion.

**Fig. 1:**
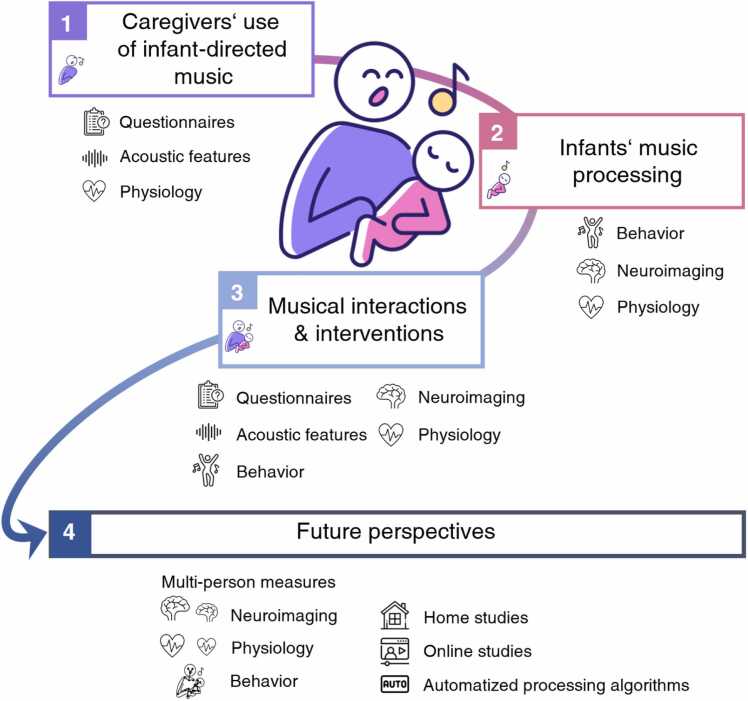
Schematic outline of the review. Section 2 focuses on the caregiver’s use of infant-directed (ID) music as evidenced by questionnaire, acoustic and physiological measures. Section 3 describes early music processing in infants assessed by behavioral, neural, and physiological measures. Section 4 details current research on early musical interactions and interventions combining measures from Section 2 and Section 3. In Section 5, we propose combining state of the art paradigms and novel analytic tools, such as multi-person measures, home and online studies, as well as automatized processing algorithms to decode early musical interactions.

## 2 Spotlight on caregivers: infant-directed (ID) music

Throughout history and across cultures, caregivers have used music to interact with their children (Custodero et al., 2003). The use of music as a tool for infant-parent bonding has been found to be an integral part of daily family life (Trehub et al., 1997), and it continued to be a prevalent practice even during the Covid-19 pandemic (Steinberg et al., 2021). According to Custodero and colleagues (2003), 60% of US-American families incorporate daily musical interactions into their routine with their infants, toddlers, and preschool-aged children, while 32% engage in such activities weekly, with only 8% not engaging in musical interactions at all within a week. This highlights the pervasive nature of music in caregiving.

ID singing is the primary musical activity mothers use when interacting with infants in their early years (Ilari, 2005). ID singing is an effective tool for promoting social engagement and bonding between parents and their children, particularly during preverbal stages when language communication is not yet possible. Research has shown that parents utilize ID singing to help regulate their own and their child's affective state, as well as to promote social connection (Papoušek, 1995; Trevarthen, 1999; Steinberg et al., 2021; Trehub, 2019). Further, ID singing is an intuitive and accessible means for parents to excite or calm their infants without requiring any formal musical training.

Overall, the ubiquity of music in early social communication and bonding between parents and their children is evident from these findings. Musical interactions between caregivers and children have been prevalent throughout history and continue to be relevant today, with ID singing being a particularly popular tool for parents.

### 2.1 ID singing: communicative functions and acoustic features

ID singing is recognizable across cultures (Hilton et al., 2022, Mehr et al., 2019, Trehub et al., 1993a) and is generally characterized as having a higher pitch, a more loving tone of voice, longer inter-phrase pauses, and slower tempi compared to non-ID singing (Trainor and Hannon, 2013). ID singing can be further categorized into two different song types used in different caregiving contexts: playsongs and lullabies (Rock et al., 1999, Trainor, 2006, Trehub and Trainor, 1998). These types of ID singing are proposed to have different communicative functions, and caregivers vary their singing performance style to convey these different communicative functions (Cirelli et al., 2020). For example, caregivers often use playsongs to engage and excite infants, potentially eliciting their attention toward relevant information. On the other hand, lullabies are intended to calm and soothe infants and are often used to put infants to sleep. Note that the distinction between lullabies and playsongs relies only partly on their musical structure and words but depends greatly on how they are “performed”. As such, the same song can be used as both a lullaby and a playsong depending on how it is rendered (Rock et al., 1999, Trainor, 2006). Interestingly, the frequency with which caregivers choose to sing in different ID styles might vary somewhat across cultures. For instance, Trehub and colleagues (1993b) reported that English-speaking caregivers often sing playsongs while Hindi-speaking mothers more typically sing lullabies. Despite this cultural variation, playsongs and lullabies share several acoustic features that make them identifiable as ID songs.

ID playsong and lullaby singing styles can be distinguished based on three perceptual domains that relate to their different functions, namely pitch height (related to the fundamental frequency, varying from low to high), loudness (related to sound intensity), and rhythm (related to tempo and patterns of sound durations and intervals) (Hilton et al., 2022, Nakata and Trehub, 2011, Trainor et al., 1997). Compared to lullabies, playsongs are characterized by higher pitch, greater pitch variability, faster rates of pitch change, more variability in those rate changes, a wider pitch range, a faster tempo, higher rhythmicity, as well as more variability of vowel space change, and lower inharmonicity (Rock et al., 1999). The exaggerated pitch contours and brilliant tone of playsongs most likely capture infants’ attention. On the other hand, lullabies are characterized by lower loudness levels and relatively more intensity at lower frequencies resulting in an airy voice quality (Trainor et al., 1997). Even though these acoustic features were initially exclusively studied in Western music samples, a recent paper found that these acoustic features of ID songs are similar across 20 different societies (Hilton et al., 2022). Moreover, mothers and fathers seem to make common acoustic adjustments when singing to their infants (Trehub et al., 1997). Taken together, these findings support the notion that music serves as a universal communication tool, underscoring the necessity for further investigation into the role of music in early developmental communication.

### 2.2 Socio-emotional effects of ID singing

ID singing and music serve communicative functions that include emotional information. When adults were asked to judge audio excerpts of mothers’ singing, they rated those excerpts as more *emotional* when directed at an infant (Milligan et al., 2003, Rock et al., 1999). Mothers also smile more when singing than speaking to their infants (Trehub et al., 2016), suggesting that ID singing is more emotionally expressive. This emotionality likely relates to caretaking goals. For example, mothers often sing in a lullaby style when soothing an upset infant, while they sing in a playsong style when attracting their infant’s attention to the mother’s face and/or interesting things in the environment, such as toys. The temporal or rhythmic structure is likely critical for the attentional and emotional power of ID singing (Trainor and Marsh-Rollo, 2019). Caregivers typically provide visual rhythmic signaling during ID singing such that women’s eye-widening and blinking are time-aligned with the rhythm (metrically strong moments) of their ID singing, and infants’ looking to the woman’s eyes also align with the rhythm (i.e., coordination; Lense et al., 2022). Thus, the rhythms of ID singing enable rich social-communicative interpersonal engagement and potentially interpersonal synchronization of brain and behavior (Markova et al., 2019, Wass et al., 2020). We outline how such dynamic adaptive interactions can be studied using state of the art approaches such as caregiver-child hyperscanning in Section 5.

### 2.3 Regulatory and bonding effects of ID singing

ID singing is not a one-way communication from the caregiver to the infant. Rather, ID singing also affects caregivers and their bond with their children. Singing is reported to increase caregivers’ positive affect and decrease their negative affect and anxiety (Fancourt and Perkins, 2018). Indeed, lullabies are suggested to help caregivers self-regulate their emotions and those of their children (Trehub and Trainor, 1998). Beyond the regulatory effects of ID singing on caregivers, music-making likely facilitates the social connection between caregiver and child (Savage et al., 2021). In general, when adults move in sync to music with others, it increases social affiliation, trust, and cooperation between participants (Savage et al., 2021), even in 14-month-old infants (Cirelli et al., 2014, Cirelli et al., 2014, Cirelli et al., 2016). Rocking or bouncing an infant while singing to them likely affects caregivers and infants similarly. Across a number of studies, parents report that singing increases feelings of closeness and attachment to their child (Creighton et al., 2013, Fancourt and Perkins, 2018, Vlismas et al., 2013). In addition, joint musical engagement of parents and children has been shown to increase affective attachment and engagement during play between dyads (Vlismas et al., 2013). Corroborating these effects, during the Covid-19 pandemic, parents also reported increased parent-child attachment as they engaged more often in musical activities (Steinberg et al., 2021). These findings support social bonding as an important function of caregivers’ and infants’ early musical interactions (Trainor and Cirelli, 2015, Savage et al., 2021). While singing per se can be related to all the aforementioned socio-emotional effects on caregivers, we suggest that these effects could also be related to information transferred from the children back to the caregivers during musical interaction. It is, therefore, essential to understand how infants *process* music (Section 3) and how caregiver-infant dyads or infant peer groups *interact* in musical contexts (Section 4).

## 3 Spotlight on children: early music processing

Research over the last few decades has revealed that music is a prevalent and important stimulus for infants (Bower et al., 2021, Trainor, 2015, Trainor and Hannon, 2013, Trehub, 2003, Trehub et al., 2015, Trehub et al., 1993, Trehub and Hannon, 2006, Trehub and Trainor, 1990). Here we examine the early development of musical pitch (Section 3.1) and timing (Section 3.2) perception and then consider how they impact early communication and socio-emotional development (Section 3.3 & Section 3.4). Perception of pitch and time, both for individual sounds and patterns of sounds, is essential for music and language processing (Bolinger, 1986, Goswami, 2011) and infants are already developing sensitivity to these acoustic dimensions before birth. Furthermore, processing rhythmic and prosodic information, which involves both pitch and timing dimensions, is crucial for language acquisition as it enables the segmentation of streams (Trainor and Adams, 1996) into meaningful chunks (Menn et al., 2022). Further, timing-related processing is suggested to be especially important for interpersonal behavioral coordination (Trainor and Cirelli, 2015) and proto-conversations (Gratier et al., 2015; V. Nguyen et al., 2022). As infants learn to infer the rhythmic patterns of (musical) event sequences, they also learn to coordinate their own behavior accordingly (Provasi et al., 2014, Zentner and Eerola, 2010). And finally, pitch and timing provide essential information for sharing emotional states in caregiver-child coordination, which is one of the primary communicative goals of early musical interactions (Shenfield et al., 2003).

### 3.1 Pitch

People perceive pitch on a continuum from low to high. Sounds perceived to have a pitch (including vocalizations and many musical instruments) typically have a physical structure with energy at a fundamental frequency, f_0_ (corresponding to the perceived pitch), and harmonics at integer multiples of f_0_ (Tramo et al., 2005). The integration of harmonics into a single sound percept is accomplished in the auditory cortex after the cochlea and subcortical nuclei have analyzed the frequency content of the incoming auditory stimulus (Bendor and Wang, 2006). That pitch is derived in the brain is evident in that a sound containing the harmonics of an f_0,_ but with no actual energy at f_0_, is perceived to have a pitch corresponding to the missing f_0_ (Moore and Gockel, 2012, Trainor, 2008).

Robust infant pitch perception is evident by 3–4 months of age when infants are able to construct the pitch of the missing fundamental (He and Trainor, 2009). For sounds containing the fundamental, pitch discrimination is evident even in newborns and improves over the first year after birth as measured using both behavioral (Saffran et al., 2005, Trehub et al., 1985), hemodynamic and electrophysiological methodologies (Choudhury and Benasich, 2011, Háden et al., 2009, Hämäläinen et al., 2011, He et al., 2007, He et al., 2009, Muenssinger et al., 2013, Stefanics et al., 2009).

Individual tones in music and phonemes in speech do not carry much inherent “meaning”, i.e., they are not necessarily interpretable or convey (much) communicative or social information. Rather, meaning arises from sequences or patterns of tones or phonemes or, in the case of music, from simultaneous combinations of tones that form chords. Infants are sensitive to many features of pitch patterns (Jentschke et al., 2008, Krumhansl and Keil, 1982, Trainor, 1997, Trainor and Trehub, 1992a, Trainor and Trehub, 1993a, Trainor and Trehub, 1993b, Trehub et al., 1986, Trehub et al., 1990), including distinguishing consonant and dissonant chords (Guerrero Arenas et al., 2016, Masataka, 2006, Schellenberg and Trainor, 1996, Weiss et al., 2020, Zentner, 1996) and changes in a single note in a pitch pattern or melody (Chang and Trehub, 1977; Dehaene-Lambertz et al., 2010; Homae et al., 2012; Plantinga and Trainor, 2009; Tew et al., 2009; Trehub et al., 1985, Trehub et al., 1987). Different musical systems around the world use different scales (division of the octave into a discrete set of intervals or notes, e.g., the Western major scale; Indian ragas), and some, including Western music, combine notes into chords and string chords into sequences forming harmonic progressions according to rules, such as those of Western tonality. Sensitivity to musical system-specific pitch structures appears to depend on familiarity and experience (Cohen et al., 1987, Weiss et al., 2020) and takes several years to develop. Young Western infants are not sensitive to Western scale structure or Western tonality in general, but rudimentary sensitivity to tonality develops after one year of age in children taking music classes (Gerry et al., 2012). Sensitivity to Western harmonic rules develops in toddlerhood and becomes more adult-like by preschool age (Trainor and Trehub, 1994; Corrigall and Trainor, 2014, Corrigall and Trainor, 2019; Jentschke et al., 2014; Virtala et al., 2013).

As discussed above, caregivers tend to sing to infants using a high-pitched voice. Accordingly, infants prefer higher pitches and high- over lower-pitched singing (Trainor and Zacharias, 1998). They also show enhanced neural mismatch negativity (an event-related potential elicited by odd stimuli within a sequence) when detecting pitch deviants in the higher voice of polyphonic music in comparison to pitch deviants in the lower voice (Marie and Trainor, 2013, Marie and Trainor, 2014), as do adults (e.g., Fujioka et al., 2005).

In sum, infants show robust pitch perception in single tones and tone patterns from early on and become more sensitive to musical system-specific pitch structures as they mature. As well, infants generally show a preference for high-pitched voices in music and speech.

### 3.2 Timing

Musical events unfold rapidly, and listeners must segment (or group) them into relevant chunks in real time to extract their meaning. Music typically contains temporal regularities that make this possible. In particular, listeners can extract a regular (usually isochronous) beat from the rhythmic surface, which typically contains a variety of sound event intervals. That the beat is constructed in the brain is evident in that listeners can perceive beats during rests or silences in musical patterns and, in some circumstances, can even perceive a beat at a frequency (tempo) for which there is no energy in the stimulus (Tal et al., 2017). Further, two or three beats at one temporal level are typically grouped together at a higher temporal level to form a metrical hierarchy in which the frequency (or tempo) of adjacent levels typically stand in 1:2 or 1:3 ratios.

Infants show such timing-related processing from early on. They are sensitive to changes in the temporal structure of rhythms (Baruch and Drake, 1997; Chang and Trehub, 1977; Cheour et al., 2002; Demany et al., 1977; Háden et al., 2015; Pickens and Bahrick, 1995, Pickens and Bahrick, 1997; Thorpe et al., 1988; Thorpe and Trehub, 1989; Trehub and Thorpe, 1989) or inconsistencies in the phrasal structure of temporal patterns (Jusczyk and Krumhansl, 1993, Krumhansl and Jusczyk, 1990, Patel et al., 1998, Trainor and Adams, 2000). Further, infants show the best tempo discrimination of Western music at a beat rate of about 600 ms, similar to adults (Baruch and Drake, 1997), suggesting an innate basis for the range of tempos over which music is constructed. EEG studies show that infants’ brains track beat and meter frequencies of auditory patterns (Cirelli et al., 2016, Winkler et al., 2009) as well as ID songs (T. Nguyen et al., 2023) and nursery rhymes (Attaheri et al., 2022). Remarkably, even premature infants at 30–33 weeks’ gestation track both beat and meter frequencies in auditory patterns (Edalati et al., 2023). Infants can also be primed to perceive a metrically ambiguous pattern – that is, a pattern that could be perceived to have a duple or a triple meter – in one meter or the other by bouncing them on either every second or every third beat (Phillips-Silver and Trainor, 2005). Further, priming by intensity accents on either every second or third beat leads to different neural responses to that same ambiguous pattern presented without accents (Flaten, et al., 2022). Taken together, these results demonstrate an early sensitivity to the temporal beat and hierarchical metrical structure of music.

Children’s temporal processing skills improve dramatically with age as they start to show similar neural patterns as in adults (Cirelli et al., 2014, Overy et al., 2004, Putkinen et al., 2013, Saffran et al., 1999, Shahin et al., 2010, Yamazaki et al., 2018). However, the particular metrical structures used in the music of different cultures vary considerably (Jacoby and McDermott, 2017, Polak et al., 2018), and rhythm development is greatly influenced by the particular musical environment in which the child is situated. Complex meters, for which one level of the metrical hierarchy is not isochronous, are common in many cultures. For example, beat groupings of 7 (4 + 3) are common in Balkan music. Hannon and colleagues (Hannon and Johnson, 2005, Hannon and Trehub, 2005a, Hannon and Trehub, 2005b) showed that Western infants readily process Balkan and Western meters at 6 months of age but that Western 12-month-olds and adults are much better with Western meters. Furthermore, it seems that this enculturation can be accelerated through music classes in infancy (Cirelli, Spinelli et al., 2016; Gerry et al., 2010). Thus, as with pitch structure, young children become specialized at processing the rhythmic structures in their musical culture both through exposure and training (Hannon and Trainor, 2007).

### 3.3 Attentional effects of ID music

Attentional processes play a crucial role in infant music processing. They are closely intertwined with other cognitive and perceptual processes, such as memory, language, and emotion. Interestingly, infants seem to pay particular attention to ID music. Infants and newborns listen longer to ID songs than adult-directed songs or even ID speech (Tsang et al., 2017). Corroborating the behavioral research, musical stimuli also produce greater hemodynamic responses in the right planum temporale in the infant's brain compared to speech stimuli (Dehaene-Lambertz et al., 2010). The right planum temporale is an area thought to be involved in audio processing, so this result highlights the saliency of music in an infant’s environment. Further, infants are particularly sensitive to positive affect in this singing as they look longer to hear ID songs sung in a “loving” tone (Trainor, 1996), even for hearing infants born to deaf parents (Masataka, 1999). Similarly, for unfamiliar songs and speech stimuli, 4- to 10-month-old infants listen longer to the happier versions (i.e., those expressing more positive affect; Corbeil et al., 2013). Additionally, 6-month-old infants look longer, and move less, in response to their mothers’ ID singing compared to ID speech, perhaps reflecting greater attentional engagement (Nakata and Trehub, 2004). Infants are also sensitive to the different types of ID songs, particularly lullabies, and playsongs (for reviews see Trainor and Marsh-Rollo, 2019; Trainor and Hannon, 2013). For example, 6-month-olds direct their attention inward for lullabies versus outward for playsongs (Rock et al., 1999). Again, familiarity appears to be important (Kragness et al., 2022, Mehr et al., 2016, Prabhakar et al., 2018), particularly for playsongs, with infants attending more to faster versions of unfamiliar foreign playsongs compared to culturally familiar ones (Conrad et al., 2011). Interestingly, infants’ attention seems to be guided by the rhythm of the music. For example, when watching videos of women singing to them, infants increase their visual attention towards the eyes of the singer in rhythmic coordination with the beats of the music (Lense, Shultz et al., 2022). Taken together, ID music seems to guide infants’ attention, with behavioral and neuroimaging research showing its saliency in eliciting infants’ attention. In addition, there is emerging research highlighting infants’ more specific attention to the rhythm of the music. For further information on infants’ rhythmic movement to music, refer to Info box 1.

Info Box 1 **Early involvement of the motor system in processing musical rhythm.**

**Table:** 

In adults, both auditory and motor brain areas are involved in processing musical rhythms (Fujioka et al., 2011, Grahn and Brett, 2007, Patel and Iversen, 2014). Notably, links between auditory and motor systems for timing are already seen in infancy, including spontaneous whole-body movements in response to music (Cirelli et al., 2020, Ilari, 2015, Nakata and Trehub, 2004, Provasi et al., 2014, Zentner and Eerola, 2010). Even though their movements are not yet precisely synchronized to the beat, infants show temporal flexibility such that their movements become faster with faster tempo music (Kragness et al., 2022, Rocha and Mareschal, 2017, Zentner and Eerola, 2010). Other characteristics of rhythms also influence infants’ and children’s movements. For example, changes in rhythmic complexity, such as during syncopation (i.e., accentuation of metrically weak temporal positions), seem to influence children’s urge to move to the music (a psychological state referred to as “groove”; Janata et al., 2012; Kragness, Anderson et al., 2022). More precisely, Cameron et al. (2022) found that children aged 3–6 years preferred medium over low syncopation rhythms for dancing, as do adults. Children only start to show clear synchronized movements to rhythms at around 4–5 years of age and especially so if they received musical training (Drake and Palmer, 2000). Beyond generating movements to the music, infants are also often moved by others, such as when they are bounced or rocked in an interaction with an adult. In this context, infants appear to integrate rhythmic sounds and (other-generated) movements even before they have the motor capacity to precisely synchronize with a beat (Cirelli et al., 2017, Phillips-Silver and Trainor, 2005, Rocha et al., 2021). This research suggests that opportunities for and experiences of auditory-movement synchrony are prevalent in infancy, including both self- and other-generated movements.

### 3.4 Regulatory effects of ID music

Beyond attentional processes, infants also appear to associate musical stimuli with emotional cues and use music as a means of communication. By five months of age, infants are able to discriminate basic emotions expressed in music, such as happiness and sadness, an ability that becomes more robust at nine months (Flom et al., 2008, Schmidt et al., 2003). By 4–6 years, children are able to infer more abstract concepts from music, such as emotional states and referential meaning; for example, they can consistently and appropriately associate different musical excerpts with different animals, likely, at least in part, on the basis of emotional cues in the music (Trainor and Trehub, 1992b). In sum, infants are able to derive simple emotional concepts from music from early on and improve their emotion recognition in music towards preschool age.

Beyond recognizing emotions in music, the use of music listening for affect regulation (i.e., self-regulation) is widespread in adolescence and adulthood (Finnerty et al., 2021, Trehub et al., 2015). Infants are particularly limited in their affect regulation skills and count on co-regulation with caregivers while building their own repertoire for self-regulation (Feldman, 2003, Wass et al., 2019). ID singing appears to be an important tool for such co-regulation that caregivers around the world use, and infants react sensitively to its emotional content. The regulating effect of ID singing is seen in that it can delay infants’ onset of distress (Corbeil et al., 2016), and infants seem to relax more to lullabies compared to non-lullaby songs, even when songs are unfamiliar (Bainbridge et al., 2021). Thus, music has profound regulatory effects on infants, and overall, infants pay special attention to ID singing and respond to the broad emotional content of ID music.

## 4 Infant-caregiver musical interactions

Throughout the first years after birth, infants and children rarely listen to music by themselves but usually experience music through social interactions (Ilari, 2005). To understand the full impact of music on development in infancy, it is therefore necessary to examine such musical interactions in the context of caregiver-infant dyads. Musical interactions include most prominently ID singing, but also joint movements, drumming and music-making with toys (Ilari, 2005, Kirschner and Tomasello, 2010, Mendoza and Fausey, 2021; Politimou et al., 2019). Here we discuss how the partners in such interactions adapt to each other and how this impacts the co-regulation of arousal (Section 4.1), engagement (Section 4.2), movement (Section 4.2), and prosocial behaviors (Section 4.3). Finally, we briefly discuss clinical interventions based on musical interactions (Section 4.4).

### 4.1 Effects of musical interactions on caregiver-infant co-regulation of arousal

As outlined earlier, caregiver-infant interactions are often structured around ID singing (Hilton et al., 2022; Trainor and Hannon, 2013; Trainor and Marsh-Rollo, 2019; Trehub, Unyk et al., 1993b). However, as discussed above, lullabies and playsongs serve different functions and have different effects on caregiver-infant arousal. For example, one study found that when mothers sang lullabies to their infants, both mother and infant arousal decreased together over time, whereas for playsongs, arousal was maintained at a high level (Cirelli, Jurewicz et al., 2020). Further, following a distressing situation where the parent was momentarily unresponsive (i.e., still-face paradigm), ID songs from the parent reduced infant skin conductance more than speech (Cirelli and Trehub, 2020). At the same time, infants showed slightly higher arousal, more positive affect, and greater attention to the parent during familiar songs compared to unfamiliar songs, suggesting that infants remember songs and engage more readily in the context of familiar songs. Though further research is needed, these studies support the idea that caregivers use singing both to regulate their own arousal levels as well as to help infants to regulate their state, likely through coordinated adaptations, such that lullabies tend to decrease, and playsongs tend to maintain arousal.

### 4.2 Effects of musical interactions on engagement & movement

Live ID singing has been shown to be significantly better than recorded music in engaging infant attention (de l’Etoile, 2006). In natural interactions between mothers and their 4-month-olds, the more the dyad engaged in spontaneous, playful singing, the more synchronized the mother’s and infant’s gaze and affect (smiling) across the interaction (Markova et al., 2020). In a live music concert involving singing and musical instruments with caregiver-infant dyads as the audience, 6- to 18-month-old infants’ attention, affect, and movement were modulated by the song style, their caregivers’ attention and level of engagement, and the infants’ own musical experience (Kragness et al., 2023). In another concert of ID performances, infant audience members’ attention and heart rates synchronized more with those of other infants during live compared to prerecorded shows (Kragness, Eitel et al., 2022). These studies suggest that live music experience, whether between a dyad or in a larger social group, enhances coordination of arousal and attention. The results need to be replicated and expanded to examine how specific signals such as movements, gestures, smiling, gaze, and musical features of the music influence coordination and affect developmental trajectories.

### 4.3 Effects of musical interactions on prosocial behavior

The importance of rhythmic coordination for social development is perhaps more obvious in older infants as they can engage directly in social behaviors - such as helping - that are beyond the capabilities of younger infants. For example, fourteen-month-old infants are more likely to help a stranger (and friends of the stranger) if the stranger had just bounced in synchrony with the infant (i.e., bouncing at the same rate, either in-phase or anti-phase) than if they bounced out-of-sync with them (Cirelli et al., 2014, Cirelli et al., 2014, Cirelli et al., 2016, Trainor and Cirelli, 2015). While synchronous bouncing without music is enough to elicit infants’ prosocial behavior, infants helped more quickly and showed more positive affect in the presence of music compared to nature sounds (Cirelli et al., 2017). And of course, music, with its regular and therefore predictable underlying beat structure, provides an ideal stimulus for coordination between people. Infant helping is also influenced by other musical factors. For example, infants also helped a stranger more when the stranger sang a song the infant was familiar with (Cirelli and Trehub, 2018) and 18-month-olds were more likely to pass an out-of-reach object to an experimenter if she first performed a song in a happy sounding way, compared to in a sad sounding rendition (Siu and Ho, 2022). Together these findings show that shortly after their first birthday, infants use cues of rhythmic coordination, familiarity, and emotional expressivity in musical, social interactions to inform their social behaviors.

Such prosocial effects are also seen in toddlers and preschoolers engaging in joint music-making. For example, 4-year-old peers were more likely to help and cooperate with each other after a joint game that included coordinated music-making compared to a joint non-musical game (Kirschner and Tomasello, 2010). A study of musical interactions in home environments found that musical play is common for 2- to 4-year-old siblings at home, and the more siblings engaged in musical play together, the more prosociality (i.e., helping, sharing, or comforting) they showed to each other (Cirelli, Peiris et al., 2020). In a sample of over 3000 Australian children, the frequency of musical activities with an adult family member at 2–3 years of age predicted prosocial skills, numeracy, and attentional regulation at 4–5 years, even after correcting for effects sociodemographic factors and reading activities (Williams et al., 2015).

The social benefits of engaging in joint musical behaviors are also seen earlier in infancy. For example, compared to passive music classes, 6-month-olds in a joint music-making class with their parent showed more advanced social development at 12 months (Gerry et al., 2012, Trainor et al., 2012), as well as larger and earlier evoked responses to piano tones, and enhanced enculturation to Western tonal structures (Trainor et al., 2012). Relatedly, mothers who enrolled in lullaby-based antenatal classes reported less newborn crying and colic, as well as higher social bonding and lower maternal stress, compared to mothers who completed the antenatal classes without lullaby instructions (Persico et al., 2017). Together these studies show the developmental benefits of music in structured and spontaneous caregiver-infant interactions.

### 4.4 Musical interventions

Given the power of music for affect regulation and social bonding, interest in music therapy interventions with infants at risk and premature infants is growing. Premature infants in the neonatal intensive care unit are physically stressed and do not experience the rhythmic movement and exposure to the mother’s heart beat they would in the womb. A meta-analysis in 2016 included 14 experiments that used music therapy in the neonatal intensive care unit (Bieleninik et al., 2016). The studies used heterogeneous methods – for example, some used recorded music while others used live music; in some, the parent was involved in the treatment and in others not – making generalization difficult. However, across studies, music therapy significantly reduced infant cardiorespiratory rate and improved infant sleep (Arnon et al., 2006, Garunkstiene et al., 2014, Hodges and Wilson, 2010, Loewy et al., 2013). Music therapies may also help the parent by reducing parental stress and anxiety and seem most beneficial when songs are chosen by the parent (Loewy et al., 2013). Thus, while studies vary in methodology and findings, we can conclude that music has therapeutic potential for premature infants and their caregivers. In addition, live music with personal meaning may have greater benefits than recorded or generic music.

Music therapy in the context of musical play between children and their caregivers has also been used in populations of older children with social interaction difficulties –particularly in those with autism – with the aims of fostering healthier social development, emotion regulation, and parent-child bonding, as well as building connections with other families (Crawford et al., 2017, Lense et al., 2022, Lense and Camarata, 2020). For example, during interactive book reading, although caregiver-toddler dyads involving children with ASD showed less visual attention and interpersonal coordination compared to dyads involving typically developing children, both groups showed greater inter-dyadic visual attention coordination when the stories were sung (musical) compared to spoken (non-musical) (Liu et al., 2022). Thus, music can aid in coordination between caregivers and young children with ASD, although more research is needed to address heterogeneity of needs, interests, and competencies among dyads.

Overall, music has potential therapeutic effects for behavioral, social, and health outcomes for premature infants and young children with ASD, their caregivers, and for social bonding between children and caregivers. Musical interventions hold promise for early development in other domains as well, such as pain management and outcomes for children with dyslexia and other developmental disorders, but a full discussion of early musical interventions is beyond the scope of the present paper.

## 5 Future perspectives: decoding early musical interactions

As highlighted in previous sections, musical interactions occur daily in family life and across different cultures. Caregivers provide multi-sensory, yet highly structured and predictable information, through music, be it via singing or instrumental music-making (see Section 2). ID music is positively received by infants, who display early developing pitch and timing skills as well as beginning abilities to use music to engage socially and emotionally (see Section 3). A growing body of literature now integrates child and caregiver perspectives to understand how adaptive musical interactions between caregivers and children support early development and well-being (see Section 4). Relying on infants’ early developing musicality, caregivers use musical behavior to promote social bonding and establish social interactions between adults and babies. Specifically, by singing songs in ways that adapt to their infant’s cues, parents can create a shared experience with their babies, helping to build trust, attachment, and emotional connections. Taken together, the above observations suggest that music could be seen as an early-emerging form of interpersonal communication, anticipating verbal communication. This suggestion triggers a fundamental question about the nature of such communication, namely, what information precisely is being communicated during such early musical interactions?

Recent theoretical debates highlight that there are many possible answers to questions concerning the content and functions of interactions involving ID singing (Mehr et al., 2021, Savage et al., 2021) that are not necessarily mutually exclusive. Unlike language, music does not convey concrete or factual meaning (unless accompanied by lyrics), and it is not necessarily an intentional form of communication in which the sender expects the receiver to cognitively interpret the meaning in a specific manner (Keitel et al., 2013, Schlenker, 2017). Yet, music can serve as an accessible aid for caregivers to communicate and express certain emotions and engage in joint actions. Thus, we can think of musical interactions between caregivers and infants as an emotional communication system through which the intention is to directly induce affect and modulate arousal in the other. This communication is mediated through sensorimotor channels and interpreted on an emotional level according to features such as rhythmic complexity and melodic and harmonic structure, as well as performance features such as tempo, tempo variability, pitch height, and voice or instrument timbre.

An important aspect of this communication is sensitivity to the cues of the other. For example, the emotion expressed by a parent at a particular time might or might not resonate with that of the infant depending on their own current internal state, leading to a complex, rich, and situated social exchange. Earlier in development, the parent might direct the infant more than vice versa, helping the infant to regulate their state. For example, playing upbeat music (as in a playsong) could distract an unhappy infant and encourage engagement in a playful context. Similarly, a caregiver might try to soothe a tired infant by singing a lullaby, through which the infant might turn their attention inward for self-regulation. Importantly, as infants mature, their perception or interpretation of the emotional messages likely improves; as well, as they mature, infants become more competent at signaling their emotional state and needs, and they can take a more active role in directing musical interactions with caregivers. One important theme through these speculations is that the power of music in early development likely relies on complex, mutually adaptive interactions between infants and caregivers. How can we learn more about this communication system through the lens of developmental cognitive neuroscience?

A growing body of research has started examining this issue from a bio-behavioral perspective with a focus on how infants perceive and respond to music made for them (Cirelli and Trehub, 2020, Kragness et al., 2022, Lense et al., 2022, Nguyen et al., 2020, Nguyen et al., 2023). However, a truly holistic approach to such dyadic or group interaction is lacking. Specifically, what is missing is a characterization of the bidirectional information transfer between the infant and the caregiver: Musical communication is not purely unidirectional, and infants often actively respond to music makers (see Raz and Saxe, 2020), thereby shaping how the music is made or sung for them, and eventually even the internal state of the caregiver. Hence, future research will need to address the participatory nature of musical interactions. Accordingly, we propose that to truly understand early musical interactions, we need to conduct multi-person studies, including state of the art approaches aimed at measuring behavior and (neuro)physiological signals from multiple individuals simultaneously. In addition, we also propose that it is important to include ecological home studies (or at least online studies conducted in participants’ homes) in order to really understand the prevalence, importance, and nature of musical caregiver-infant interaction in the real world. Together, these approaches can pave the way to understanding early musical interactions.

### 5.1 Beyond the individual: multi-person behavior and neuroscience

Only a few studies have examined bidirectional information transfer in the context of early musical interactions. The emerging evidence highlights that musical interactions between caregivers and children are not only driven by the caregivers. Instead, infants actively participate by producing salient behaviors such as rhythmic movements, gaze, and vocalizations that might, in turn, affect how caregivers musically interact with them (Lense et al., 2022, Zentner and Eerola, 2010). In what follows, we discuss a number of recent methodological, technological, and analytical developments that might empower a sound and scientific study of musical communication in early childhood.

#### 5.1.1 Multi-person brain measurements

We advocate using state of the art neuroimaging techniques such as hyperscanning (Markova et al., 2019, Wass et al., 2020). Hyperscanning implies simultaneously recording brain activity from two or more individuals while they interact with each other (Montague et al., 2002), including in the context of naturalistic interactions, such as learning a song or playing a game (Gugnowska et al., 2022; T. Nguyen et al., 2020; T. Nguyen, Schleihauf et al., 2021; Pan et al., 2021; Piazza et al., 2020; Reindl et al., 2018; Santamaria et al., 2020). Notably, previous developmental studies have shown that it is feasible to conduct hyperscanning using electroencephalography (EEG) or functional near-infrared spectroscopy (fNIRS) to study (non-musical) communication during parent/adult-child interactions (Endevelt-Shapira et al., 2021, Leong et al., 2017; T. Nguyen, Abney et al., 2021; Piazza et al., 2020).

Specifically addressing musical interactions, hyperscanning recordings have the potential to shed light on two interesting neurophysiologically grounded phenomena. First, when two or more people engage in a social context, their brain activity *synchronizes* (Czeszumski et al., 2020, Dumas et al., 2010, Hoehl et al., 2020). Interestingly, such synchrony has been related to socio-cognitive processes, such as interaction and relationship quality and emotion regulation (T. Nguyen et al., 2020; T. Nguyen, Schleihauf et al., 2021; Reindl et al., 2018). A common criticism in the field, though, is that being in a similar environment and hearing the same music might induce a certain amount of similarity in parents' and infants’ brain responses. To mitigate this issue, specific control conditions or causal approaches need to be considered (Gugnowska et al., 2022, Moreau and Dumas, 2021, Novembre and Iannetti, 2021a, Novembre and Iannetti, 2021b, Wass et al., 2020). Second, hyperscanning recordings might be used to characterize *information flow*, for example, by computing to what extent the brain activity of the caregiver predicts upcoming activity in the brain of an infant and, crucially, how the brain or behavior of the infant might predict brain activity of the caregiver. Hence, these variables might index the degree to which two agents are engaged in an interaction and exchanging information in a unidirectional or bidirectional manner (Marriott Haresign et al., 2022, Wass et al., 2020). Additionally, hyperscanning can be used to investigate in both infants and caregivers the neural mechanisms underlying social, emotional, and cognitive aspects of musical experiences, such as empathy, shared attention, and behavioral coordination. This approach can help us understand how music can support social and emotional development in infants and how it can facilitate parent-child bonding.

#### 5.1.2 Multi-person behavioral and physiological measurements

Beyond hyperscanning, research on synchrony and information flow during musical interactions in early childhood can also benefit from behavioral and physiological measurements. We know little about the real-time dynamics of behavioral and physiological coordination and information flow during parent-child musical communication, but studies of movement interactions between performing musicians show that greater information flow between their movements relates to the quality of their performances (A. Chang et al., 2017; D’Ausilio et al., 2012, D’Ausilio et al., 2015) and that synchrony increases while information flow decreases as musicians learn to play a piece together (Bishop et al., 2019, Wood et al., 2022). In terms of development, two studies highlight the feasibility to concurrently measure multiple levels of coordination using fNIRS hyperscanning and dual-electrocardiography in parent-child interactions (Nguyen et al., 2021, Reindl et al., 2022). These studies show that neural and physiological synchrony seem to diverge during parent-child interaction, suggesting they are measuring different aspects of the interactions, and further highlighting the need for a more holistic picture of the processes underlying early social interactions. Regarding early musical interactions, interesting questions to be asked include how caregivers and infants move together to music as well as how are they able to make music together.

#### 5.1.3 Accessible and controlled music-making tasks

While investigating how caregivers and infants engage in joint music-making is intriguing, the process of simultaneously measuring their brain activity poses methodological challenges. The dynamic nature of joint music-making requires sophisticated techniques to capture the intricate coordination and synchronization between participants. Additionally, the simultaneous measurement of brain activity from caregiver-infant dyads introduces methodological considerations such as signal interference. In particular, movement (especially mouth movement) artifacts can strongly compromise the quality of neuroimaging recordings, especially EEG. This makes it difficult to measure reliable data from participants engaged in singing or vocalizing. Mitigation strategies include using imaging methods such as fNIRS, which are less affected by mouth movements, and conceiving music-making tasks that do not rely on mouth movements. For instance, infants can use percussion-based instruments that permit them to produce rhythmic (proto-musical) sounds using e.g., rattles and drums (Kirschner and Tomasello, 2010, Laudanska et al., 2022, Yu and Myowa, 2021), and these types of musical behaviors could be extended to dyadic interactions. Future research should investigate the functional significance of these behaviors, notably whether and when they serve communicative functions, which could be done by including measures of mutual attention as well as engagement through gaze and other modalities (Feldman, 2007).

Still, instrumental music-making, notably including both rhythm and melodies, is largely accessible to trained participants only. However, recent technological innovations have led to several devices that allow laypeople to produce music, or musical sounds, without undergoing training. One of these innovations is the E-music box (Novembre et al., 2015), a digital instrument that permits everyone to make music through movement. The E-Music box transforms cyclical rotatory movements into a musical melody whose tempo varies according to the velocity of the rotation. In a similar vein, Kragness and Trainor (2018) had young children repeatedly tap a key to produce each note or chord in a piece of preprogrammed music to understand how they would use timing and intensity to convey, for example, emotion in musical production (Kragness and Trainor, 2018). Crucially, these methodologies have the potential to be used in interactive situations involving young children and non-musically trained adults, and could be paired with EEG and other physiological or behavioral measures. They could also be extended to interactions between preschool-aged peers. Hence, such devices can turn music-making into an accessible and highly controlled activity to support the exploration of the cognitive, behavioral, and neural processes underlying musical communication. As a recent example, Novembre and colleagues (2019) showed that adult participants’ empathic abilities predicted how well dyads rhythmically synchronized while collectively making music on the E-Music box. Future studies might extend this approach, for example by providing users with control over other musical dimensions besides tempo, such as pitch, especially considering the literature on early pitch processing reviewed above (Section 3).

#### 5.1.4 Handling noisy datasets

Neural data from infants and children tend to be noisy. However, several guidelines now offer support on processing and analyzing parent-child fNIRS (Nguyen, Hoehl et al., 2021) and EEG (Kayhan et al., 2022, Marriott Haresign et al., 2022, Turk et al., 2022) hyperscanning data. Furthermore, there has been a noteworthy flourishing of general preprocessing recommendations for infant EEG (Gabard-Durnam et al., 2018, Lopez et al., 2022), as well as tutorials and toolboxes that can instruct naïve users on how to extract measures of coordination, neural tracking, synchrony, and information flow (Ayrolles et al., 2021, Jessen et al., 2021). Most likely, hyperscanning analysis techniques for dealing with developmental data will continue to improve over the coming years. Overall, we suggest that hyperscanning can provide a unique perspective on the impact of music on the brain and social behavior and offer new insights into how music can support early development and parent-child interactions.

#### 5.1.5 Beyond the caregiver

In addition to dyadic studies, the field would benefit from multi-person studies involving individuals other than the primary caregiver, particularly beyond infancy when children interact more with peers. Notably, recent studies have highlighted that children benefit and learn much from their peers (Piazza et al., 2021). Piazza and colleagues specifically showed that when children successfully learned new words together, they also showed similar brain activities. A similar approach could therefore be utilized to assess how large groups of children process music collectively during live performances (Kragness et al., 2022, Kragness et al., 2023). Studying additional multi-person settings, such as with siblings or in a daycare context, might also deepen our understanding of how infants process music in a social context.

To conclude, we believe that decoding musical interactions in early childhood requires going beyond research in individuals and assessing how infants perceive and interact with music in social contexts with others. By utilizing multi-person recordings, we can gain insights into the dynamic processes underlying bidirectional and adaptive music-making between infants and others. Despite the notable methodological challenges discussed above, a growing number of studies have shown that multi-person neural, behavioral and physiological measurements in early social interactions are feasible. Furthermore, emerging devices such as the e-music box might mitigate measurement artifacts during naturalistic musical interactions.

### 5.2 Beyond the lab: home and online studies

As outlined in previous sections, most studies on early musical interactions have been conducted in a laboratory with highly controlled stimuli presented in unnatural settings. These controlled studies have increased our fundamental understanding of infants’ and children’s musicality and will continue to do so in the coming years. However, future research should also consider experiments conducted in naturalistic environments (e.g., Kragness et al., 2022), including children's homes, when feasible. This might be particularly important to test whether the results of lab-based studies hold true outside of the laboratory. Likewise, research conducted outside of the laboratory in the first instance, like online studies, should be followed up and verified in the laboratory under highly controlled conditions and potentially using neurophysiological methods that are extremely difficult with online studies. Notably, coinciding with the Covid-19 pandemic, which forced laboratory research to pause, home and online studies increased dramatically and revealed the potential of online research to study infants and children in their real-world contexts (Steinberg et al., 2021).

#### 5.2.1 Home studies

Home studies were common in developmental psychology research a few decades ago (Hart and Risley, 1992, Hops et al., 1987), but have reemerged in recent years along with new technologies for collecting complex real world data (Costa-Giomi and Sun, 2016, Mendoza and Fausey, 2021). Two main approaches have emerged so far: either infants´ acoustic environment is sampled through microphones, or data is collected from parent-reports. Using the former approach, Mendoza and Fausey (2021) assessed day-long recordings of infants’ sound space at home. They showed that nine percent of everyday experiences were musical, resulting in infants accumulating on average about 1600 h of musical experience in their first year after birth. This research approach is important for informing future studies on early musical enculturation, notably by providing a description of the kind of musical material infants encounter across the early months. Other home studies have used parents’ extensive at-home observations and videos to show, for instance, that 90% of infants produce recognizable dancing behavior by their first year (Kim and Schachner, 2022). This finding nicely exemplifies how behaviors that might be difficult to elicit in an unfamiliar lab setting, such as spontaneous dancing, can be examined through home studies (Tervaniemi, 2023). Related to new technologies, it should be noted that home studies can now sometimes include neurophysiological measures by employing wearable devices made for mobile infant data collection, such as heart rate (Wass et al., 2019, Wass et al., 2022), eye tracking (Pérez-Edgar et al., 2020) or neural activity (Hölle et al., 2021, Park et al., 2018, Stangl et al., 2023). The addition of these measurements has the potential to greatly increase our understanding of infants’ and children’s musical perception and interaction in ecologically valid social contexts.

#### 5.2.2 Online studies

The refinement of online developmental data collection spurred by the COVID-19 pandemic has the potential to greatly augment and inform laboratory-based studies. For example, online eye tracking can be comparable to lab-based eye-tracking in some cases (e.g., Bánki et al., 2022). Current online studies in the music cognition domain include examining children’s synchronized dancing to music with various levels of syncopation (Cameron et al., 2023). Online studies have several advantages, including the possibility of collecting large datasets and access to populations of infants and children from different cultures around the world.

#### 5.2.3 Handling rich data sets

Even though home and online studies yield highly interesting results, analyzing and interpreting the data comes with challenges. The datasets can be large as studies become more accessible to parents from different locations (Sheskin et al., 2020), so processing this amount of data using conventional approaches such as micro-coding (also called manual labeling) might prove challenging. Micro-coding has been the go-to method for developmental research for many years (e.g., Beebe et al., 2010; Feldman, 2003), but it requires many hours of laboriously coding of each video frame. A potential solution is using video-based automatized analysis of behavior, which allows for complex feature extraction with fewer resources needed. Examples include analysis of multi-person body posture (e.g., Openpose), facial analysis (e.g., Openface), and eye tracking (e.g., Koul et al., 2023, Baltrusaitis et al., 2018; Cao et al., 2021; Jongerius et al., 2021). Recently such techniques have been increasingly applied to developmental data (Rocha and Addyman, 2022; Solby et al., 2021). A notable example, originally developed for animal models, is DeepLabCut, one of the various algorithms that uses deep learning to automatically track the movements of people in videos (Mathis et al., 2018). The software works by training a deep neural network to recognize the position of specific body parts of a person or features of an object and to track them over time.

In sum, we suggest that home and online studies, especially in combination with neurophysiological and automatized video-based measures, will help elucidate the socio-cognitive processes underlying early musical interactions by enabling large sample sizes and data collection in naturalistic real-world settings.

## 6 Conclusion

The literature reviewed here highlights the significance of music in early childhood development and its importance as a communicative tool between caregivers and infants. We find that caregivers readily and intuitively use ID music to soothe or engage infants. On the other side, infants readily perceive and respond to the different communicative functions of ID music. Previously, these different research angles have been studied largely independently, but we argue that understanding how caregivers and infants mutually adapt to each other using music is critical for understanding early development. We review recent studies on musical interaction that offer the opportunity to take a novel, holistic approach to studying social communication through music in early childhood. Thus far, it has been shown that musical interactions between caregivers and infants enhance co-regulation and prosocial behavior, thereby supporting infants' social and emotional development. However, the underlying neural, physiological, and behavioral mechanisms of musical interactions in early childhood are not well understood. More research is needed to elucidate these mechanisms and their impact on child development. Rather than focusing on singular parts of a complex picture, a holistic approach could help integrate different research angles and uncover the underlying mechanisms of musical interactions. Accordingly, we need to engage in multi-person neuroscience, including investigating the relations between different measures and mechanisms, including at neural (e.g., EEG, fNIRS), physiological (e.g., heart rate, skin conductance), and behavioral (e.g., movement, eye gaze, facial expression, vocalizations) levels. These studies should be supplemented by home and online investigations to examine the generalization of lab findings to real world environments and diverse populations and cultures, and by new analytical tools, such as those used in recent human adult or animal research, to investigate the dynamic nature of musical interactions between caregivers and infants. Overall, such studies will lead to greater understanding of why caregiver-infant musical interactions are universal across cultures as well as their consequences for development. Understanding the underlying neural, physiological, and behavioral mechanisms could also lead to more effective interventions supporting infant development and well-being.

## Declaration of Competing Interest

The authors declare that they have no known competing financial interests or personal relationships that could have appeared to influence the work reported in this paper.
